# Oral and Dental Considerations in Pediatric Leukemic Patient

**DOI:** 10.1155/2014/895721

**Published:** 2014-03-04

**Authors:** Chiyadu Padmini, K. Yellamma Bai

**Affiliations:** ^1^Department of Pedodontics and Preventive Dentistry, Malla Reddy Institute of Dental Sciences, Hyderabad, Andhra Pradesh, India; ^2^NTR University of Health Sciences, Vijayawada, Andhra Pradesh, India; ^3^Department of Pedodontics and Preventive Dentistry, Mally Reddy Women's Dental College, Hyderabad, Andhra Pradesh, India

## Abstract

Throughout the world, there have been drastic decline in mortality rate in pediatric leukemic population due to early diagnosis and improvements in oncology treatment. The pediatric dentist plays an important role in the prevention, stabilization, and treatment of oral and dental problems that can compromise the child's health and quality of life during, and follow up of the cancer treatment. This manuscript discusses recommendations and promotes dental care of the pediatric leukemic patients.

## 1. Introduction

Leukemia constitutes approximately 30% of all childhood cancers and acute lymphoblastic leukaemia (ALL) is the most common type of malignancy encountered in children [1, 2]. ALL accounts for about 75% of childhood leukemias with its peak incidence at 4 years of age [3]. Acute lymphoblastic leukemia (ALL) is a malignant disorder resulting from the clonal proliferation of lymphoid precursors with arrested maturation [4]. The disease can originate in lymphoid cells of different lineages, thus giving rise to B-cell or T-cell leukemias or sometimes to mixed lineage leukemia. Acute lymphoblastic leukemia was one of the first malignancies to respond to chemotherapy [5]. Among various leukemia categories, it was the first leukemia that could be cured in a majority of children [6]. Since then, much progress has been made, not only in terms of treatment but also in prevention of oral problems due to cancer treatment in children.

The most common signs and symptoms of ALL are anorexia, irritability, lethargy, anemia, bleeding, petechiae, fever, lymphadenopathy, hepatosplenomegaly, and bone pain and arthralgias caused either by leukemic infiltration of the perichondral bone or joint or by leukemic expansion of the bone marrow cavity leading to disability in walking in children [3]. The most common head, neck, and intraoral manifestations of ALL at the time of diagnosis are lymphadenopathy, sore throat, laryngeal pain, gingival bleeding, and oral ulceration [7]; see Table 1.

## 2. Methods

The current paper revision included a new systematic literature search of the PubMed electronic data base using the following parameters: terms: “pediatric cancer,” “pediatric oncology,” “hematopoietic cell transplantation,” “bone marrow transplantation,” “mucositis,” “stomatitis,” “chemotherapy,” “radiotherapy,” “acute effects,” “long-term effects,” “dental care,” “oral health,” and “pediatric dentistry.”

## 3. The Oncology Treatment of Acute Lymphoblastic Leukemia Is Divided into Four Phases [8]

*(1) Remission Induction.* It generally lasts for 28 days and consists of 3 or 4 drugs (examples are vincristine, prednisone, and L-asparaginase), with a 95% success rate. Achievement of remission is a known prerequisite for prolonged survival.

*(2) CNS Preventive Therapy/Prophylaxis.* CNS can act as a sanctuary site for leukemic infiltrates because systemically administered chemotherapeutic drugs are not able to cross the blood-brain barrier. Cranial irradiation and/or weekly intrathecal injection of a chemotherapeutic agent, usually methotrexate, are used. This presymptomatic treatment can be done in each phase as well.

*(3) Consolidation or Intensification.* It is designed to minimize the development of drug cross-resistance through intensified treatment, in an attempt to kill any remaining leukemic cells.

*(4) Maintenance.* It is aimed at suppressing leukemic growth through continuous administration of methotrexate and 6-mercaptopurine. The optimal length of this phase has not been established yet but usually lasts from 2.5 to 3 years.

The patient's blood counts normally start falling five to seven days after the beginning of each cycle of chemotherapy and/or radiotherapy staying low for approximately 14 to 21 days before rising again to normal levels for a few days until the next cycle begins [8]. A multidisciplinary approach involving oncologists, nurses, dieticians, pediatric dentists, and other related health professionals is essential in caring for the child before, during, and after any cancer therapy [9].

Ideally, all dental care should be completed before cancer therapy is initiated, and if it is not feasible, priority treatments should be eradicating acute infectious sources like extraction of grossly decayed teeth, periodontal diseases, root canal therapy for permanent teeth, and replacement of faulty restorations to avoid oral tissue irritation [15]. The oral mucosa is highly susceptible to the effects of chemotherapy and radiotherapy due high mitotic activity and is the most frequently documented source of sepsis in the immunosuppressed pediatric leukemic patients. For these reasons, early and definitive dental intervention, including comprehensive oral hygiene measures, reduces the risk for oral and associated systemic complications [15].

The dental treatment for leukemic children can be broadly divided into 3 phases [8]:

 phase 1: dental and oral care before the initiation of cancer therapy,
 phase 2: dental and oral care during immunosuppression periods,
 phase 3: dental and oral care after the cancer therapy is completed.

### 3.1. Phase 1: Dental and Oral Care before the Initiation of Cancer Therapy

The objectives of a dental/oral examination before cancer therapy starts are stated below [16]:

1. to identify and stabilize or eliminate existing and potential sources of infection and local irritants in the oral cavity without needlessly delaying the cancer treatment or inducing complications. Any existing lesions that might normally lie dormant can flare up and become life threatening once the child is immune suppressed;
2. to educate the patient and parents about the importance of optimal oral care in order to minimize oral problems and discomfort before, during, and after treatment and about the possible acute and long-term effects of the therapy in the oral cavity and the craniofacial complex; see Table 6.

Initial evaluation of oral and dental examination includes

1. reviewing the child medical history,
2. reviewing current hematological status,
3. reviewing the proposed chemotherapy/radiation protocol,
4. completing a thorough head, neck, and dental examination including panoramic and bitewing radiographs.

Medical history review includes disease/condition (type, stage, and prognosis), treatment protocol (conditioning regimen, surgery, chemotherapy, radiation, and transplant), medications (including bisphosphonates), allergies, surgeries, secondary medical diagnoses, hematological status (complete blood count (CBC)), coagulation status, immune suppression status, and presence of an indwelling venous access line. Most of the leukemic patients have a central line, which is an indwelling catheter inserted into the right atrium of the heart, which is useful for obtaining blood samples and administering chemotherapeutic agents. The American Heart Association (AHA) recommends that antibiotic prophylaxis for non valvular heart devices is indicated only at the time of placement of these devices in order to prevent surgical site infections [17].

#### 3.1.1. Hematological Considerations

*(1) Absolute Neutrophil Count (ANC).* ANC > 1,000/mm^3^: there is no need for antibiotic prophylaxis [15]. However, few authors suggest that antibiotic coverage dose (as per American Heart Association recommendations [18]) may be prescribed when the ANC is between 1,000 and 2,000/mm^3^ [19]. If infection is present or unclear, more aggressive antibiotic therapy may be indicated and should be discussed with the medical team.

ANC < 1,000/mm^3^: elective dental care should be deferred until the ANC rises. In case of dental emergency, discussion should be done with medical team regarding antibiotic coverage beyond endocarditis prophylaxis before proceeding for dental treatment.

*(2) Platelet Count [15].* Platelet count > 75,000/mm^3^: there is no need of additional support but the* *dentist should be prepared to treat prolonged bleeding by using sutures, hemostatic agents, pressure packs, gelatin foams, and so forth.

Platelet count is 40,000 to 75,000/mm^3^: platelet transfusions may be considered pre- and 24 hours postoperatively. Localized procedures to manage prolonged bleeding may include sutures, hemostatic agents, pressure packs, and/or gelatin foams.

Platelet count < 40,000/mm^3^: elective dental care should be deferred. In dental emergency cases, discussion should be done with patient's physician to discuss supportive measures (e.g., platelet transfusions, bleeding control, and hospital admission) before proceeding.

Meticulous oral care is important to reduce the incidence and severity of oral sequelae of the treatment protocol. Parents and child should be educated about the importance of good oral hygiene practices throughout the entire oncology treatment, regardless of the child's hematological status [20].

A regular soft toothbrush or an electric brush used at least twice daily is the most efficient means to reduce the risk of significant bleeding and infection in the gingiva [21]. Periodontal problems should be eliminated primarily because chemotherapeutic treatment could stimulate myelosuppression, and during myelosuppression period chronic periodontal situations could show an acute attack [22]. Leukemic children who present poor oral hygiene or periodontal disease are prescribed chlorhexidine rinses [19]. Mouth rinses containing alcohol should be avoided because they can dry the mucosa and aggravate mucositis. Sodium bicarbonate or saline mouthwash 3 or 4 times a day could also be recommended for these patients. Sodium bicarbonate (5%) could dilute the mucous secretion, moisten the oral mucosa, increase the oral pH, and inhibit the *Candida albicans* colonization [23].

The leukemic child may be at high risk for dental caries from a dietary standpoint for a number of reasons as patients are prescribed daily nutritional supplements rich in carbohydrate to maintain or gain weight and oral pediatric medications contain high amounts of sucrose (e.g., nystatin) which makes them prone for dental caries. So fluoride supplements and neutral fluoride rinses or gels are indicated for those patients who are at risk for caries. Use of 0.12% chlorhexidine mouthwash twice daily has been found to effectively suppress the major pathogens present in the oral cavity [24]. Teeth with poor prognosis (e.g., impacted teeth, root tips, partially erupted third molars, teeth with periodontal pockets > 5 mm, teeth with acute infections, and nonrestorable teeth) should be removed ideally 3 weeks before cancer therapy starts to allow adequate healing and special attention to be given to extraction of permanent teeth in patients who will receive or have already received radiation to the face as it poses a risk of osteoradionecrosis [25, 26]. All surgical procedures must be as atraumatic as possible, with no sharp bony edges remaining and satisfactory closure of the wounds [27]. A platelet count of 50,000/mm^3^ is advisable for minor surgeries like simple extractions, whereas a minimum level of 100,000/mm^3^ is desirable for major surgeries like removal of impacted teeth [28]. The local hemostatic measures must be considered such as pressure packs, sutures, use of a gelatin sponge, and topical thrombin or microfibrillar collagen.

Fixed orthodontic appliances can harbor food debris, compromise oral hygiene, and act as mechanical irritants, increasing the risk for secondary infection. Removable appliances and retainers must be advised when tolerated by the patient who shows good oral care. Dahllöf et al. described the following strategies to provide orthodontic care for pediatric leukemic patients [29]:

1. usage of appliances that minimize the risk of root resorption,
2. use of lighter forces,
3. terminating treatment earlier than normal,
4. choosing the simplest method for the treatment needs,
5. not treating the lower jaw.

### 3.2. Phase 2: Dental and Oral Care during Immunosuppression Periods

#### 3.2.1. Objectives [16]

The objectives of a dental/oral care during cancer therapy are stated below:

1. to maintain optimal oral health during cancer therapy,
2. to manage any oral side effects that may develop as a consequence of the cancer therapy,
3. to reinforce the patient and parents' education regarding the importance of optimal oral care in order to minimize oral problems/discomfort during treatment.

Acute manifestations that develop during immune suppression are mucositis, gingival bleeding, xerostomia, secondary candidiasis, and herpes simplex and bacterial infections. Mucosal ulceration is the most frequent oral problem encountered and is associated with low neutrophil counts and the aim of oral management is to relieve symptoms and to prevent and treat any secondary infection [30].

#### 3.2.2. Clinical Significance of Mucositis

Oral mucositis can be very painful condition and can significantly affect nutritional intake, mouth care, and quality of life of leukemic child [31]. Oral mucositis typically arises within two weeks after initiation of chemotherapy and agents such as antimetabolites and alkylating agents cause a higher incidence and severity of oral mucositis in children [32]. Oral mucositis initially presents as erythema of the oral mucosa that often progresses to erosion and ulceration. A white fibrinous pseudomembrane typically covers the ulcerations and lesions heal within approximately 2–4 weeks after the last dose of stomatotoxic chemotherapy or radiation therapy [33].

Several factors affect the clinical course of mucositis. In chemotherapy-induced oral mucositis, lesions are usually limited to nonkeratinized surfaces (i.e., lateral and ventral tongue, buccal mucosa, and soft palate) [34]. In radiation-induced oral mucositis, lesions are limited to the tissues in the field of radiation with nonkeratinized tissues affected more often. The clinical severity is directly proportional to the dose of radiation administered [35]. A study conducted by Elting et al., demonstrated that patients with oral mucositis are significantly more likely to have severe pain and a weight loss of ≥ 5% compared to controls [36].

In a study conducted by Trotti et al., they proved that approximately 16% of patients receiving radiation therapy for head and neck cancer were hospitalized due to mucositis and among them 11% of the patients had unplanned breaks in radiation therapy due to severe mucositis [37]. Thus, oral mucositis is a major dose limiting toxicity of radiation therapy that in turn compromises treatment plan and its outcome.

Maintenance of good oral hygiene reduces the severity of mucositis. A study done by Da Fonseca proved that those patients who do intensive oral care have a reduced risk of developing moderate/severe mucositis and its associated complications like infections and septicemia [38].

#### 3.2.3. Assessment Scales for Oral Mucositis [39]

Mucositis severity should be graded using a validated scale, such as the WHO scale, prior to initiating supportive therapy, to establish a baseline and to assess the treatment outcome.

The WHO scale describes the degree of soreness, erythema, and ability of the child to have normal diet. Patients with grade I and grade II mucositis should brush their teeth as described earlier with a soft toothbrush and fluoride toothpaste and rinse with a salt and bicarbonate solution.

With grade III mucositis, children should be advised to clean the oral cavity 4 times a day as described earlier or with gauze dipped in a salt and bicarbonate solution. The salt and bicarbonate solutions should be used every 4 to 6 hours if possible. Grade IV mucositis requires the patient to cleanse the oral cavity 4 times a day with either a soft toothbrush or gauze and to use a salt and bicarbonate solution every 4 hours.

The Cancer Institute scale describes the severity of pain, change in oral intake, and death related to mucositis.

Oral Assessment Guide reviews the extent of mucositis in greater depth in the pediatric population compared to WHO and NCI scales; see Tables 1, 2, and 3.

#### 3.2.4. Oral Care Protocols [39]

A standard first step to prevent mucosal injury is the implementation of good oral hygiene and the use of a standardized oral care protocol for all children undergoing chemotherapy. Various authors in literature have studied a number of oral care protocols.

Cheng et al. conducted a study on the effectiveness of an oral care protocol for the prevention of chemotherapy-induced oral mucositis in children. The protocol was evaluated over an 8-month period in 42 pediatric cancer patients who ranged in age from 6 to 17 years.

The results obtained from this study were significant and demonstrated a 38% reduction in the incidence and severity of ulcerative mucositis along with decrease in pain in the experimental group who were instructed to follow proper technique of toothbrushing, 0.2% chlorhexidine mouth rinse, which was used twice a day, and a 0.9% saline rinse, which was used in the morning, after each meal and before going to bed [40].

Costa et al., evaluated 14 children, whose ages ranged from 2 to 10 years. Oral health protocol began at least 1 day before initiating the chemotherapy and ended 10 days after the end of this period. The average length of treatment was 8 weeks. The treatment group consisted of 7 patients who received a mouth rinse with a nonalcoholic solution of 0.12% chlorhexidine and oral hygiene care, including twice-daily toothbrushing. The control group followed the same protocol for brushing their teeth but was given a placebo mouth rinse. The results were considered statistically significant. Only 1 child from the treatment group, compared to 5 children in the control group, developed oral mucositis. The oral lesions were less severe and of a shorter duration in the children who received chlorhexidine mouth rinses [41].

#### 3.2.5. Clinical Management of Oral Mucositis [33]

A variety of nonpharmacological and pharmacological agents have been used in practice for the prevention and management of oral mucositis in children. There is no gold standard at this time, as there is a lack of evidence-based recommendations. So research should be focused to bring a gold standard treatment protocol for oral care in children. Multinational Association for Supportive Care in Cancer and the International Society of Oral Oncology (MASCC/ISOO) have developed clinical practice guidelines for the management of mucositis.

Management of oral mucositis in children is divided into the following sections:

1. pain control,
2. nutritional support,
3. oral decontamination,
4. palliation of dry mouth,
5. management of oral bleeding and therapeutic interventions for oral mucositis.

*(1) Pain Control [33].* The pain associated with mucositis significantly affects nutritional intake, oral care, and quality of life of child. The use of saline mouth rinses, ice chips, and topical mouth rinses containing an anesthetic such as 2% viscous lidocaine relieve pain.

The stepwise approach that should be followed is as follows:

1. beginning with oral rinses (saline solution, sodium bicarbonate rinses, etc.),
2. topical anesthetics (lidocaine and benzocaine),
3. mouthwashes combination (“magic mouth rinse” containing diphenhydramine, lidocaine, and combinations of aluminum hydroxide; magnesium hydroxide; and simethicone),
4. mucosal surface protectants such as hydroxypropyl cellulose gels or sucralfate solutions.

When these medications do not provide adequate relief, a step-up approach to systemic analgesics is warranted; see Table 4.

*(2) Nutritional Support [33].* Nutritional intake can be severely compromised by the pain associated with severe oral mucositis and taste changes occur due to chemotherapy and/or radiation therapy that limits food intake [39]. In patients expected to develop severe mucositis, a gastrostomy tube is sometimes placed prophylactically. Guidelines published by the American Society for Parenteral and Enteral Nutrition state that parenteral nutrition should be considered in children who cannot maintain adequate nutritional intake orally or enterally for 5 to 7 days. But the potential risks of parenteral nutrition, including increased risks of infection, electrolyte abnormalities, and cholestatic liver disease, must be considered [42].

*(3) Oral Decontamination [39].* The microbial colonization of oral mucositis lesions exacerbates the severity of oral mucositis and decontamination of oral cavity will reduce the severity of mucositis [10, 39]. Patients who have developed severe oral mucositis also have been found to be three times more likely to develop bacteremias resulting in increased length of hospital stays as compared to patients without mucositis.

*Chlorhexidine [39].* Chlorhexidine mouthwash is effective antimicrobial compound and topical prophylactic agent against oral mucositis and candidiasis. It acts as a local anesthetic and anti-inflammatory agent that possesses no antimicrobial activity. Cheng and Chang conducted a randomized 2-period crossover study that compared the efficacy of 0.15% benzydamine and 0.2% chlorhexidine mouthwashes in alleviating the symptoms of oral mucositis in children undergoing chemotherapy. Of the 34 patients who were evaluated, 26% of the chlorhexidine group compared to 48% of the benzydamine group showed WHO grade II mucositis. The results revealed a significant difference in mouth pain and decreased difficulty in eating/chewing and swallowing in favor of chlorhexidine [43].

*(4) Palliation of Dry Mouth [33].* Patients undergoing cancer therapy often develop transient or permanent xerostomia and hyposalivation, which further aggravate inflamed tissues, increase risk for local infection, and make mastication difficult.

The following measures can be taken for palliation of a dry mouth:

1. sipping water as needed to alleviate mouth dryness. Several supportive products including artificial saliva are available;
2. rinsing with a solution of 1/2 teaspoon of baking soda (and/or 1/4 or 1/2 teaspoon of table salt) in 1 cup warm water several times a day to clean and lubricate the oral tissues and to buffer the oral environment;
3. chewing sugarless gum to stimulate salivary flow;
4. using cholinergic agents as necessary.

*(5) Management of Bleeding [33].* Local intraoral bleeding can usually be controlled with the use of topical hemostatic agents such as fibrin glue or gelatin sponge.

#### 3.2.6. Advanced Therapeutic Interventions for the Treatment of Oral Mucositis in Children

*(1) Cryotherapy [33].* Topical administration of ice chips to the oral cavity during administration of chemotherapy results in decreased delivery of the chemotherapeutic agent to the oral mucosa. This effect is due vasoconstriction and reduced blood flow. Studies conducted by Aisa et al., and Mahood et al. have stated that cryotherapy reduces the severity of oral mucositis in patients receiving bolus doses of chemotherapeutic agents [44, 45]. The disadvantage of cryotherapy is that it is only useful for short bolus chemotherapeutic infusions and do not have a role in radiation-induced oral mucositis.

*(2) Growth Factors [33].* Reduction in the proliferative capacity of oral epithelial cells is thought to play a role in the pathogenesis of mucositis, so various growth factors that can increase epithelial cell proliferation have been studied for the management of oral mucositis. A study conducted by Von Bültzingslöwen et al. showed that IV recombinant human keratinocyte growth factor-1 significantly reduced incidence of oral mucositis in patients with hematologic malignancies receiving high-dose chemotherapy and total body irradiation before autologous hematopoietic cell transplantation [46]. Intravenous human fibroblast growth factor-20 is currently in clinical development for reduction of mucositis secondary to high-dose chemotherapy in autologous hematopoietic cell transplant patients [47].

*Anti-Inflammatory Agents [33].* Benzydamine hydrochloride is a nonsteroidal anti-inflammatory drug that inhibits proinflammatory cytokine TNF-*α*. In one phase III clinical trial, benzydamine hydrochloride mouth rinse reduced the severity of mucositis in patients with head and neck cancer undergoing radiation therapy of cumulative doses up to 50 Gy radiation therapies [48].

*(3) Antioxidants.* A topical oral applicant, RK-0202, consists of the antioxidant N-acetylcysteine. In a placebo-controlled phase II trial in patients with head and neck cancer, this agent significantly reduced the incidence of severe oral mucositis up to doses of 50 Gy radiation therapies [49].

*(4) Glutamine [39].* During times of stress, including cancer, glutamine stores decrease in body by over 50%, contributing to the development of oral mucositis [50].

Use of supplemental glutamine has been said to regulate gastrointestinal cell growth, function, and regeneration. A study done by Skubitz and Anderson found that glutamine decreased the duration of mucositis to 4.5 days compared with placebo. Glutamine lessened the pain associated with mucositis, specifically pain that altered eating habits [51]. Glutamine has no taste and is suspended in a sucrose vehicle, so it will be palatable to children.

*Oral Sucralfate Suspension [39].* Sucralfate is used for gastric ulcer prophylaxis and treatment. Its mechanism of action is not clear, but it is believed that sucralfate binds to ulcerations, creating a protective barrier to aid in healing [52]. Shenep and colleagues conducted a study in 48 children and adolescents with newly diagnosed acute nonlymphocytic leukemia. The treatment group who used sucralfate suspension had showed 58% bacterial colonization rate compared to 92% in control group. No oral pain was reported in 58% of patients receiving sucralfate, compared to 25% receiving placebo [53].

*(5) Palifermin [39].* As a recombinant keratinocyte growth factor, palifermin stimulates the proliferation, differentiation, and migration of epithelial cells throughout the gastrointestinal tract. A study was conducted by Vadhan-Raj et al. Eighty-eight percent of the patients receiving placebo experienced grade II or higher mucositis, compared to only 44% of the palifermin treated patients. Opioid use was also decreased in the palifermin treated group [54].

*(6) Low-Level Laser Therapy [33].* The mechanism by which LLLT accelerates wound healing in mucositis is due to the following [55]:

1. increased mitochondrial ATP production,
2. the local release of growth factors,
3. increased proliferation of fibroblasts,
4. detoxifying oxygen free radicals or reducing the formation of these free radicals,
5. increases microcirculation in mucosa.

In a study conducted by Djavid in 45 patients who underwent chemotherapy, low-level laser therapy significantly reduced the incidence and duration of grades 3 and 4 oral mucositis, decreased the risk of secondary infection, and accelerated return to normal nutrition [55].

A meta-analysis done by Clarkson et al., (2004), in a Cochrane review of 27 publications, found that, among 8 prophylactic agents used to obtain relief from mucositis, crushed ice yielded the best results [56]. Keefe et al. (2007) published an update on the clinical guides for the prevention and treatment of oral and gastrointestinal mucositis which is currently the most widely used treatment that is the use of 2% lidocaine rinses in combination with 0.12–0.2% chlorhexidine during 30 seconds, every three or four hours [57]. Colella et al., (2010) have suggested that the frequent application of a spray consisting of synthetic collagen precursor amino acids in combination with sodium hyaluronate can offer rapid and effective pain relief and contributes to mucosal healing [58].

*(7) Xerostomia [33].* High-dose irradiation to the head and neck region has been shown to cause changes in the chemical composition of the saliva as well as a reduction in the rate and volume of salivary flow in adults [59, 60]. Radiation-induced xerostomia will also cause a shift in the oral microflora to a highly acidogenic and cariogenic population [61].

A study conducted by Nasman et al. proved that a significantly greater proportion of children treated with total body irradiation or chemotherapy harbor high bacterial counts of *Streptococcus mutans* in comparison with the control children and children in the treatment group exhibited a low saliva buffer capacity [62]. Regardless of caries risk, brushing should be done twice daily with fluoridated toothpaste [63].

In addition, daily rinsing with chlorhexidine digluconate is also recommended for children with a high caries risk. Daily use of a self-applied fluoride gel is effective in patients with xerostomia to prevent dental caries. A regular periodic dental radiographic imaging is needed to monitor caries risk [51].

*(8) Trismus [33].* Daily oral stretching exercises/physical therapy must continue during radiation treatment. Management of trismus may include prosthetic aids to reduce the severity of fibrosis, trigger-point injections, analgesics, muscle relaxants, and other pain management strategies.

*(9) Osteoradionecrosis [14].* The management of osteoradionecrosis should aim to afford pain relief, control soft tissue and bone infection, and avoid or reduce the progression of bone necrosis; see Table 5.

The diagnostic criteria of osteoradionecrosis are as follows:

1. presence of one or more ulcerated mucosal lesion of the alveolar processes, with or without the exposure of maxillary or mandibular bone,
2. exposed bone presenting a necrotic appearance,
3. lesions presenting spontaneously or after dentoalveolar surgery (particularly extractions),
4. absence of healing over a period of at least 6 weeks.

### 3.3. Phase 3: Dental and Oral Care after the Cancer Therapy Is Completed [16]

The objectives of a dental/oral examination after cancer therapy ends are stated below:

1. to maintain optimal oral health,
2. to reinforce the importance of* *optimal oral and dental care for life to the patient/parents.

The patient should be seen at least every 6 months or in shorter intervals if issues such as xerostomia or trismus are present.

Patients who have experienced moderate or severe mucositis are closely monitored for any changes in oral mucosa. Orthodontic care may start or resume after completion of all therapy and after at least a 2-year disease-free survival when the risk of relapse is decreased and the patient is no longer using immunosuppressive drugs [64].

*Long-Term Effects of Oncotherapy on Pediatric Patients [65].* The treatment of leukemia severely alters odontogenesis process and the severity depends on the age of the child. The younger the child is, the greater the oral complications noticed as both dentitions, deciduous and permanent, are affected during the earlier stages of development. The most common changes are microdontia, agenesis of teeth, change in crown or root shape, defective mineralization of the dental structure, and delayed tooth eruption [66–68]. Along with change in tooth size and shape, irradiation also affects growth centers affecting maturation of craniofacial complex resulting in asymmetrical facial growth and malocclusion [69].

The degree and severity of these defects depend on the child's age at diagnosis, type of central nervous system treatment, and the dose of cranial irradiation. Children treated before 5 years of age had the most severe dental defects, suggesting that immature teeth were at greater risk for developmental disturbances than mature teeth. Many case reports and animal studies confirm this hypothesis and suggest that dental defects may be related to chemotherapy, radiotherapy, or both [65]. Study conducted by Sonis et al. in 97 children to evaluate craniofacial development in long-term survivors of acute lymphoblastic leukemia found that craniofacial effects of therapy were observed in only those children who received 2400 cGy before 5 years of age. Ninety percent of children in this group had diminished mandibular growth that was determined by cephalometric analysis [70].

Another similar study conducted by Guyuron et al., has shown that among 41 patients who received irradiation to the head and face during growth thirty-eight of them had soft tissue or bony deformities [71]. A postulated mechanism, which explains the development of altered craniofacial growth in children who underwent craniofacial radiotherapy, is due to that alteration in pituitary gland function [72–74]. Recent study done by Minicucci et al. (2003) showed delays in dental development, hypoplasia, and microdontia in children receiving chemotherapy.

## 4. Conclusion

The key to success in maintaining a healthy oral cavity during cancer therapy is patient compliance. It is very much vital to educate the caretaker and child about the importance of oral care to minimize discomfort and increase the chances for a successful outcome of oncology treatment. Discussion with caretakers and patient should include dietary habits, the potential cariogenicity of pediatric medications and nutritional supplements, and late effects of the conditioning regimen on the craniofacial growth and dental development. The participation of a pediatric dentist in the hematology/oncology team during treatment is of irrefutable importance in reducing the complications before, during, and after leukemic treatment in children.

## Figures and Tables

**Table 1:** World Health Organization (WHO) recommendations for grading of acute and subacute toxic effects [10].

Grade	Symptoms
0	None
1	Soreness/erythema
2	Erythema/ulcers/can eat solids
3	Ulcers/require liquid food
4	Alimentation is not possible

**Table 2:** “Common terminology criteria for adverse events,” version 4.0. advocated by National Cancer Institute (NCI) [11].

Grade	Symptoms
0	Asymptomatic/mild symptoms; intervention not indicated.
1	Moderate pain; no interference with oral intake; modified diet indicated.
2	Severe pain; interference with oral intake.
3	Life threatening consequences; urgent intervention required.
4	Death.

**Table 3:** Oral assessment guide [12].

Category	Numerical and descriptive ratings		
	1	2	3
Voice	Normal	Deeper or raspy	Difficulty in talking, painful
Swallow	Normal swallow	Some pain on swallow	Unable to swallow
Lips	Smooth, pink, and moist	Dry or cracked	Ulcerated or bleeding
Tongue	Pink and moist and papillae are present	Coated or loss of papillae with shiny appearance, with or without redness	Blistered/cracked
Saliva	Watery	Thick or copy	Absent
Mucous membrane	Pain and moist	Reddened or coated (increased whiteness) without ulcerations	Ulceration with or without bleeding
Gingiva	Pink, stippled, and firm	Edematous with or without redness	Spontaneous bleeding or bleeding on pressure
Teeth or dentures	Clean and no debris	Plaque or debris in localized areas (between two teeth, if present)	Plaque or debris generalized along gum line or denture bearing areas

**Table 4:** WHO analgesic ladder for the 3 stepwise treatments to relieve pain in mucositis [13].

Step 1	Nonopioid* ± adjuvant^†^	Pain persisting or increasing. Step up

Step 2	Opioid for mild to moderate. Pain ± nonopioid ± adjuvant	Pain persisting or increasing. Step up

Step 3	Opioid for moderate to severe pain^§^ ± nonopioid ± adjuvant	

*Aspirin, acetaminophen, and nonsteroidal anti-inflammatory drugs.

^†^Amitriptyline, carbamazepine, and gabapentin for neuropathic pain; prednisone or dexamethasone for pain associated with intracranial pressure, nerve, and spinal cord compression.

^‡^Codeine, hydrocodone, and tramadol.

^§^Morphine, fentanyl, and hydromorphone.

**Table 5:** Staging classification and treatment of osteonecrosis of the jaws [14].

Staging classification	Clinical manifestations	Treatment
Stage 1	Exposed bone necrosis or small oral ulceration without exposed bone necrosis, but without symptoms.	Rinses with 0.12% chlorhexidine and checkup.

Stage 2 A	Exposed bone necrosis or a small oral fistula without exposed bone necrosis, but with symptoms controlled with medical treatment.	Rinses with 0.12% chlorhexidine, antibiotic, analgesics, and checkup.

Stage 2 B	Exposed bone necrosis or a small oral fistula without exposed bone necrosis, but with symptoms not controlled with medical treatment.	Rinses with 0.12% chlorhexidine, antibiotic, analgesics, and surgery with removal of the zone of bone necrosis.

Stage 3	Jaw fractures, skin fistula, and osteolysis extending to the inferior border.	Rinses with 0.12% chlorhexidine, antibiotic, analgesics, and extensive surgery with resection of bone.

**Table 6:** Flow chart showing oral and dental care before, during, and after chemotherapy for leukemic children [14].

Treatment before chemotherapy	Treatment during chemotherapy	Treatment after chemotherapy
The dentist should consult the oncologist to determine the current condition of the patient and the type of treatment planned.	The oncologist should be consulted in order to know the degree of immune suppression of the patient.	The dentist should consult the oncologist to determine immune competence.

(i) Exhaustive examination of the oral cavity: discard periapical lesions and/or bone alterations and the evaluation of periodontal health. (ii) Denture fitting should be checked, with readjustment or removal of those prostheses that prove trauma. (iii) Radiological study: intraoral (periapical and bitewing) and panoramic.	Treatment of the complications of chemotherapy (mucositis, xerostomia).	(i) Insist on the need for routine systematic oral hygiene. (ii) Use of chlorhexidine rinses and fluorization.

General prophylactic measures: tartar removal, dental fluorization, and rinses with 0.12% chlorhexidine.	Continued patient reminder of the need to maintain strict dental hygiene is indicated, with the added use of chlorhexidine rinses and fluorization.	

The patient should be informed of the complications of treatment.	(i) Analgesics: paracetamol/metamizol. (ii) No NSAID. (iii) Antibiotics: dose adjustment is required according to the observed creatinine clearance values in patients with kidney problems.	

Teeth that are nonviable or present a poor prognosis should be removed: (1) minor surgery: at least two weeks before chemotherapy. (2) major surgery: 4–6 weeks before chemotherapy.	No elective dental treatment should be carried out. Only emergency dental care.	Elective dental treatment.
